# Action Understanding Promoted by Interoception in Children: A Developmental Model

**DOI:** 10.3389/fpsyg.2022.724677

**Published:** 2022-02-21

**Authors:** Hui Zhou, Qiyang Gao, Wei Chen, Qiaobo Wei

**Affiliations:** ^1^Center for Brain, Mind and Education, Shaoxing University, Shaoxing, China; ^2^Department of Psychology, Shaoxing University, Shaoxing, China

**Keywords:** action, interoception, action understanding, children, development

## Abstract

Action understanding of children develops from simple associative learning to mentalizing. With the rise of embodied cognition, the role of interoception in action observation and action understanding has received more attention. From a developmental perspective, this study proposes a novel developmental model that explores how interoception promotes action understanding of children across ages. In early infancy, most actions observed in infants come from interactions with their caregivers. Babies learn about action effects through automatic interoceptive processing and interoceptive feedback. Interoception in early infancy is not fully developed, such as the not fully developed gastrointestinal tract and intestinal nervous system. Therefore, in early infancy, action understanding is based on low-level and original interoceptive information. At this stage, after observing the actions of others, infants can create mental representations or even imitate actions without external visual feedback, which requires interoception to provide internal reference information. By early childhood, children begin to infer action intentions of other people by integrating various types of information to reach the mentalizing level. Interoception processing requires the integration of multiple internal signals, which promotes the information integration ability of children. Interoception also provides inner information for reasoning about action intention. This review also discussed the neural mechanisms of interoception and possible ways by which it could promote action understanding of children. In early infancy, the central autonomic neural network (CAN) automatically processes and responds to the actions of caregivers on infants, providing interoceptive information for action understanding of infants. In infancy, the growth of the somatomotor system provides important internal reference information for observing and imitating the actions of infants. In early childhood, the development of interoception of children facilitates the integration of internal and external information, which promotes the mentalization of action understanding of children. According to the proposed developmental model of action understanding of children promoted by interoception, there are multilevel and stage-dependent characteristics that impact the role of interoception in action understanding of children.

## Introduction

Understanding actionP is an important aspect of development of children. The multilevel model of action understanding proposed by Casartelli and Molteni (2014) explains that there are different and non-competitive types of action understanding, ranging from lower-level associative mechanisms to higher-level “mentalizing” abilities. Action understanding starts from simple stimulus-response association to understanding the action intention through mental processes such as cognitive reasoning. The development of action understanding of children is in line with the process of associative learning and mentalizing. For example, infants are able to automatically imitate mouth opening of someone (Meltzoff and Moore, 1977), and, by early childhood, children can speculate on the action intention of an executor (Meltzoff, 1995; Galilee and McCleery, 2016).

Interoception may play a cohesive role between action observation and understanding. Previous studies have emphasized the association between visual observation and actions of children but underestimated the role of interoception. For example, de Klerk et al. (2019) suggested that action imitation of children is formed through the coupling between perception and action. However, the disparity between the weak action ability and understanding of infants makes it difficult for simple perception-motor associations to explain the development of action understanding of children (Fotopoulou and Tsakiris, 2017). Meltzoff (2007) proposed that infants monitor their bodily acts and understand the actions of others *via* proprioception. In addition to proprioception, embodied simulation theory holds that there is a close relationship between interoception, action observation, and action understanding (Meltzoff and Moore, 1997; Freedberg and Gallese, 2007).

Interoception refers to the sense of the physiological condition of own body of an individual, such as hunger, satiety, and thirst. It includes visceroception of the heart, stomach, lung, and other viscera and proprioception of the skin, muscles, and bones (Garfinkel et al., 2015a). Interoception is present since the early years, developing rapidly during infancy (Quattrocki and Friston, 2014; Brewer et al., 2015). Although the metacognitive component of interoception continues to develop well into adolescence, other aspects (e.g., objective interoceptive sensitivity) reach maturity in childhood (Murphy et al., 2017). Indeed, interoception growth of children is a prerequisite for understanding their actions (Bowman et al., 2017; Nicholson et al., 2019). For example, the polyvagal theory suggests that there are distinct autonomic subsystems in mammals. These subsystems are related to social communication (such as facial expression) and responsive behavior (such as fight-flight behaviors and behavioral shutdown). Compared with the polyvagal theory, this study considers that the interoception processing involves not only the autonomic nervous system but also the joint processing of multiple brain regions. Action understanding of children changes with age, and thus, the role of interoception on action understanding of children may undergo changes as well.

**FIGURE 1:**
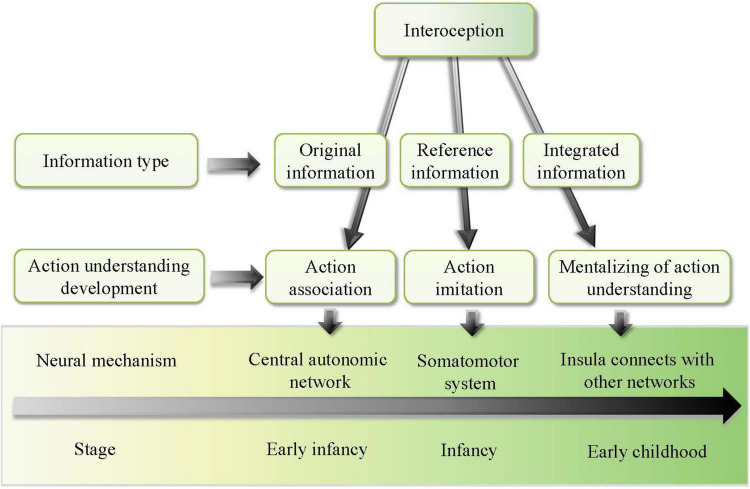
Developmental model of action understanding of children promoted by interoception.

Therefore, this study established a developmental model of action understanding of children as promoted by interoception. We argued that interoception is the main driver of action understanding in development. As shown in Figure 1, the model suggests that action understanding of children develops with age, going through different stages, including simple act association in early infancy, action observation and imitation in late infancy, and action mentalizing in early childhood. At different stages, the role of interoception in promoting the development of action understanding of children is different. This study discusses in detail how interoception promotes action understanding of children at different developmental stages.

## Interoception Promotes the Development of Children’s Action Understanding

### Action Association Originates From Interoception

Interoception has inheritable traits and develops in early infancy (Klabunde et al., 2019). Although not mature, infant interoception allows infants to interact with the external environment and others. Initial action understanding is based on interoceptive feedback about what is necessary for survival. Consider thirst as an example. At first, infants only perceive the bodily signal of thirst, but they have not experienced drinking water and receiving feedback afterward. Therefore, infants do not understand why caregivers give them water at first. Only after experiencing drinking water for some time are infants able to associate the interoception of thirst and the action of drinking water, thereby understanding the feeding action of caregivers (Harshaw, 2008). Human newborns can establish classical conditioned reflex by associating sensory stimuli with simultaneous stimuli. The simple association also is adaptive according to the feedback (Lipsitt, 1990). The classical or operant conditionings also support early association learning of infants between interoception and action. Quattrocki and Friston (2014) proposed that infants associate the interoceptive signals of warmth or fullness with caregivers, thus promoting the interaction between infants and caregivers and social development.

The actions observed by early infants are mainly directed at themselves, which allows them to understand actions through interoceptive feedback. Newborns are unable to perform most actions required for survival (e.g., eating, drinking water, and going to the toilet), thus, relying on their caregivers (Reddy and Uithol, 2016). Therefore, in early infancy, because infants do not actually experience these actions, it is difficult for them to understand actions through sensorimotor associations (Cañal-Bruland et al., 2010). Instead, when infants perceive the actions of other people, interoceptive information feedback facilitates their understanding of and responses to actions. For example, Fairhurst et al. (2014) found that infants can understand and respond to different stroking actions. When a caregiver caresses a 9-month-old baby at a moderate speed, the heart rate of the baby slows down and becomes quiet. Infants younger than 6 months of age provide feedback to their mothers if they are in an uncomfortable holding posture through crying and other behaviors (Esposito et al., 2013).

Through bottom-up interoceptive feedback, infants can even have predictive responses to the actions of others (Murphy et al., 2017). Infants as young as 2 or 3 months can respond predictably to their actions of caregivers. For example, proprioception from being held up by adults allows 2-month-old babies to adjust the stiffness of their bodies as adults reach for them (Reddy et al., 2013). An electroencephalography (EEG) study of infant somatosensation found a significant bilateral mu desynchronization over the frontocentral cortex of an infant before somatosensory stimulation. The finding suggests infant prediction of somatosensory stimulation (Shen et al., 2021). Visceroception from the stomach allows infants to open their mouths in advance in response to the sight of the caregiver with a spoon (Brisson et al., 2012). Moreover, predicting actions can further regulate interoception and promote interpersonal interactions. For instance, the gastrointestinal tract and intestinal nervous system are not fully developed at birth (Gershon, 1999), and different feeding methods (e.g., breastfeeding vs. bottle feeding) determine the feeding actions of mothers, to which infants adjust their internal state (Bramhagen et al., 2006).

In sum, during early infancy, infants observe the actions of others and understand them through interoceptive feedback. However, due to the immaturity of interoception of infants based on internal scattered physiological signals and direct association learning, action understanding of children is still limited at this stage.

### Action Imitation Based on Proprioception

Around 1–3 years of age, toddlers observe actions that not only orient them but also point to other targets. At this developmental stage, children consciously imitate an action after observing it (Subiaul et al., 2016); thus, interoception like the sense of body agency and ownership plays an important role in both action observation and imitation (Gao et al., 2019). In action observation, the mirror mapping of actions requires internal body representation (Freedberg and Gallese, 2007). The theory of embodied simulation holds that internal representation provides the processing mechanism of action observation and understanding (Cañal-Bruland, 2017). The observer establishes the corresponding relationship between the observed action and the corresponding internal representation in the brain (Rizzolatti and Sinigaglia, 2010). For example, young children correct their imitative facial responses, which suggests a visual-proprioceptive cross-modal matching process (Meltzoff and Moore, 1997; Marshall and Meltzoff, 2014). Studies have shown that action observation is robustly associated with the activation of the primary somatosensory cortex (Caspers et al., 2010), demonstrating the connection between proprioceptive and external senses, such as visual perception. The observed actions are imitated and coordinated through the ventral anterior motor cortex to understand the motion components of actions (Gazzola and Keysers, 2009).

Interoception processing of children develops from the scattered internal bodily information to its integration into a whole and unified internal representation and from unconscious interoceptive processing to conscious control (Murphy et al., 2017). This development allows action representation of children. Infants form their own body schema in the first 6 months of life, which develops rapidly during the next 2 years (Keromnes et al., 2019). Proprioception improvement provides an internal reference for children to understand the observed actions and imitate them without looking at their bodies. According to the AIM (active intermodal mapping) model of Meltzoff and Moore, infant imitation is affected by three components, namely, the body part used, the action performed, and the goal achieved (Meltzoff and Moore, 1997). Body representation is indispensable in infant imitation. For example, in an EEG study of 14-month-old infants, those who observed actions that involved the hand or foot had stronger desynchronization of the mu rhythm in the corresponding areas of the sensorimotor cortex (Saby et al., 2013).

Due to the immaturity of proprioception, it is still challenging for infants within 1 year of age to imitate and understand actions through internal representation. Infants younger than 6 months mainly focus on action production from different body parts (Reddy et al., 2013; Reddy, 2015). For 9-month-olds, it is still difficult to imitate contralateral actions because of poor contralateral representation (Boyer and Bertenthal, 2016). However, from 2 years of age, with the improvement of proprioception, children can better imitate the observed actions based on a unified internal representation. For example, Yoo et al. (2016) investigated the EEG activity of 9- and 12-month-old infants when they observed purposeful actions and performed the same actions. The results showed that 1-year-olds showed activation in their own sensorimotor regions when they observed actions, and they were able to establish a corresponding relationship between the observed actions and their own actions. At the age of 3 years, children are more proficient in proprioception and body control (Rochat and Morgan, 1995). Well-developed proprioception can help children to observe and imitate actions without being limited by specific action effectors (Caspers et al., 2010) as well as to form a holistic action understanding in the brain through internal representation.

In infancy, the rapid development of proprioception of children provides an internal reference for observing and imitating the actions of others. According to the AIM model, when observing and producing actions, children need to pay attention to the body part used in the action, performance of actions, and goals (Meltzoff and Moore, 1997). The dual attention system (Corbetta and Shulman, 2002) proposes two different attention systems. The goal-driven (i.e., top-down) attention system, which includes parts of the intraparietal cortex and superior frontal cortex, is involved in goal-directed selection for stimuli and responses. The stimulus-driven (i.e., bottom-up) attention system, which includes the temporoparietal cortex and inferior frontal cortex, is more sensitive to behaviorally relevant detailed stimuli. In the process of observing and producing actions, when children spend less of their internal resources on the action details, they will pay more attention to inferring the intention or purpose of the actions of others (Arnold et al., 2019).

### Integrated Processing of Interoception Promotes Mentalizing of Action Understanding

Interoception regulates and maintains the balance of the internal environment of the body (Sterling, 2012; Craig, 2015). It integrates multimodal sensory information, including bottom-up input signals from the body and top-down regulatory instructions from the central nervous system (Sterling, 2012; Arnold et al., 2019). Internal signals such as temperature, pain, and heart rate need to be transmitted to the central nervous system through internal sensory afferent receptors such as thermoreceptors, chemoreceptors, mechanoreceptors, and baroreceptors (Berntson and Khalsa, 2021). These interoceptive afferent nerves are widely distributed, unmyelinated, and thin nerve fibers that innervate internal sensors. Interoceptive signals are not definite, single signals (Quigley et al., 2021). For example, cardiovascular information is signaled by different sensors that encode the occurrence, intensity, blood pressure, and neurovascular afferent signals of the heartbeat (Zeng et al., 2018). Therefore, unlike visual or auditory perception, interoceptive signals conveyed by multiple sensors in a non-synchronous way need to be integrated (Petzschner et al., 2021).

Therefore, interoception processing requires the interaction between the brain and body to achieve a dynamic balance by integrating multisensory information (Bonaz et al., 2021). Interoception is controlled not only by the central autonomic neural network (CAN) (Berntson and Khalsa, 2021) but also by high-order neural networks such as the insula (Craig and Craig, 2009). The regulation of the internal state of the body by the brain needs to integrate a variety of information for recognition and inference. Taking breathing as an example, the respiratory rhythm is produced in the brainstem, usually in an unconscious state, but also through the top-down cognitive function to identify and infer the interoceptive signal, and through the emotional control network to change the internal state (Weng et al., 2021). In interoception inference, the central nervous system estimates the internal state using an approximate Bayesian inference. The central nervous system uses sensory data with various uncertainties and noises to verify the prediction model based on *a priori* in real time to estimate the internal state (Barrett and Simmons, 2015; Barrett et al., 2016).

The interoceptive processing mode has an important influence on mentalizing, such as action understanding and intention reasoning (Murphy et al., 2017). Infancy is an important stage of interoception. Numerous interoceptive signal processes, such as hunger, satiety, thirst, and muscle tension, are mainly formed during infancy (Harshaw, 2008). However, at this early stage, babies are not proficient at processing or regulating interoceptive signals and often process them in an implicit way (Murphy et al., 2017). With the improvement of interoception in children, including continuous attention and cognitive monitoring, their interoception awareness is also enhanced (Garfinkel et al., 2015b; Weng et al., 2021), allowing them to control and regulate interoception processing in a top-down manner (Klabunde et al., 2019). At the age of 4 years, interoceptive processing of children affects their reasoning and ability to predict action intentions of people.

At the age of 4 years, children are able to understand action intentions, and their action understanding reaches the mentalizing level (Ansuini et al., 2015). Inferring an action intention is challenging for children because the relationship between action and intention is not a one-to-one mapping (Csibra, 2007; Jacob, 2013), that is, the same actions may have different intentions. For example, if someone suddenly waves their hand when walking on the street, their intention may be to take a taxi or drive a wasp away (Kilner et al., 2007a). Thus, to understand action intentions of others, we made inferences based on multiple information (e.g., actions and situation). For example, through situation information, such as a taxi near the street, we can speculate the intention of a waving action (Kilner et al., 2007a); through the expression or kinematic information of an action, such as a gesture holding a cup, we can guess whether a friend wants to drink. Therefore, integrating multiple information to make inferences plays an important role in action understanding. Indeed, the predictive coding model assumes that action understanding requires the establishment of multiple prediction models based on the integration of internal and external information as well as choosing the priority model based on the principle of minimizing the prediction error (Kilner et al., 2007b; Kilner, 2011; Clark, 2013).

Action understanding, which is based on the integration and processing of various types of information, creates a model for reasoning and predicting similar to the interoception processing model. In that sense, the interoception processing model developed in the early years promotes action understanding mentalizing. Interoceptive information is also integrated into action understanding processing, providing internal information for action reasoning. For example, a study on 3–5-year-olds showed that their internal representation is closely related to their action development, having a significant predictive effect on individual differences of 3–5-year-olds in mentalizing (Meltzoff, 2013). Studies further found that only when the action external information and internal representation are fully integrated can advances in action understanding promote mentalizing of children (Bowman et al., 2017).

### The Neural Mechanisms Involved in Interoception and Action Understanding

#### The Central Autonomic Network in Action Association

Within 1 year of age, the direct feedback of low-level interoceptive information promotes the establishment of action associations of children. Low-level interoceptive information is controlled mainly by the CAN. Similar to the brainstem, the nucleus tractus solitaries (NTS) receives afferent interoceptive information from the spinal cord or vagus nerve (Berntson and Khalsa, 2021). The CAN includes the anterior cingulate forebrain, amygdala, hypothalamus, and brainstem, and it is an internal regulatory system that controls visceral movement, neuroendocrine activity, and other vital internal signals for survival (Benarroch, 1993). The CAN receives the down-up interoceptive signals and reflects automatically, allowing the individual to adapt to the changing internal or external environment and be in a stable state (Beissner et al., 2013).

Interoception is often unconscious (Bonaz et al., 2021). For example, visceral regulation is considered to be mainly composed of low-level reflexive mechanisms, which are autonomous processes (de Groat and Tai, 2015). The NTS is a typical visceral information-receiving area (Berntson and Khalsa, 2021). Although interoception is also regulated by the cerebral cortex and affects high-level information processing, studies have shown that visceral signals affect higher neurobehavioral processes (Berntson et al., 2003). However, interoception awareness in early childhood is not fully developed (Klabunde et al., 2019). Therefore, during infancy and childhood, unconscious rather than conscious interoception processing mainly promotes understanding of action associations by children.

Moreover, interoception of infants is closely associated with their behavioral interactions of caregivers. For example, when the hypothalamus of an infant detects lower-than-baseline blood glucose levels, a crying response will be elicited, prompting the caregiver to feed the infant and balance his/her internal needs (Pezzulo et al., 2015). The CAN controls and regulates interoceptive information, which is associated with the responsive actions of caregivers to promote adaptation and action understanding.

#### Somatomotor System in Action Imitation

In interoception, the sense of body agency and representation are closely related to the somatomotor system (Murata et al., 2016). Proprioception is a kind of subjective consciousness in which acts are executed by themselves. When the action is recognized as the result of own body of an individual, it produces a sense of agency (Keromnes et al., 2019). The somatomotor system in the brain not only controls itself to produce complex actions, but it is also related to the internal representation of the body. Brain imaging studies have confirmed that in healthy people, the parietal cortex is involved in action detection and proprioceptive generation (Chambon et al., 2013). When the inferior parietal cortex is damaged, the sense of agency and body representation of individuals are impaired (Keromnes et al., 2019).

Action observation and production can activate motor systems, such as the ventral premotor cortex, inferior parietal lobule, primary motor cortex (Dushanova and Donoghue, 2010), dorsal premotor cortex (Tkach et al., 2007), inferior parietal cortex (Chong et al., 2008), and other brain regions. The ventral anterior motor cortex and inferior parietal lobule are the classical mirror neuron regions for action observation (Buccino et al., 2004; Aziz-Zadeh et al., 2006). Based on proprioception, people can mirror and imitate observed actions without having to see their bodies. Therefore, researchers suggest that the mirror reaction of action observation is based on the association between observed action and proprioception (Cook et al., 2014). For example, selectively observing different finger acts can activate the potential amplitude of the area corresponding to the fingers in the motor cortex (Catmur et al., 2011).

During development, 7-month-old infants already have a rich neural representation of the body. For instance, tactile stimulation of different body parts in the infant results in similar activation patterns as adults (Saby et al., 2015; Meltzoff et al., 2018). Well-developed body representations facilitate action observation and imitation of children. Even if they cannot see their own body parts, children match their own body to bodies of others to produce imitative actions. For example, when 14-month-old infants see a head-touch act, they can imitate the act even if they cannot see their own head (Meltzoff and Marshall, 2020). After observing adult mouth action, a child automatically imitates the action without observing his/her mouth (Heyes, 2011). By establishing an association, the superior temporal sulcus (STS), which processes the visual characteristics of the act (Oram and Perrett, 1996), the parietal cortex (Gallese et al., 2002), and the ventral premotor cortex (Rizzolatti et al., 2002; Umesawa et al., 2020) integrate visual action information with the internal representation of the body to imitate and understand actions (Rizzolatti and Sinigaglia, 2010).

Therefore, it is essential for the somatomotor system to develop representation and the sense of body agency of children. The somatomotor system actively participates in the processing when observing and imitating the actions of other people.

#### The Insular Cortex Connects With Other Brain Regions

The insular cortex (IC) is the main cortical region that processes interoceptive information (Hassanpour et al., 2018), including proprioception and visceroception. Studies have shown that the IC may be the key anatomical region that integrates the internal input signals from the body (Karnath and Baier, 2010), form emotional feelings, and provide a sense of body ownership (Craig and Craig, 2009). In a functional magnetic resonance imaging (fMRI) study, participants watched a video in which an individual smelled something in a glass that produced either nausea or pleasure. The results showed that the left anterior insula and the right anterior cingulate cortex were activated to some extent by observing smelling actions of others (Wicker et al., 2003). Interoception is further processed by the IC to produce corresponding emotions and cognition, which promotes socialization and mentalizing of an individual (Devue et al., 2007).

The IC plays an important role in the development of interoception of children. Interoceptive awareness and proprioception of children develop rapidly, and the IC is an important area for interoceptive signal processing (Keromnes et al., 2019). During processing, the IC extensively connects with other subcortical and cortical regions (Gehrlach et al., 2020). After integrating top-down and bottom-up information, interoception can be adjusted or regulated in a timely manner. For example, visceral signals, such as hunger, are processed by the IC and change depending on whether people see food and have expectations (Livneh et al., 2020). For children, their physical needs are affected by the responses of their mothers and have a long-term impact on the ability of children to recognize their own internal state and emotions, which are mainly processed in the insular region (Fotopoulou and Tsakiris, 2017).

In action understanding, interoception processing in the IC not only provides internal information reference for action understanding but also connects with other brain regions to understand action intentions and even mentalizing of other people. For example, if an observer only observes and recognizes actions, an input from the motor cortex and visceral motor center is needed to establish the relationship between actions of other people and his/her own action experience. However, if the observer perceives and imitates social actions, in addition to the motor cortex, it will also activate the insular region (Casartelli and Molteni, 2014), suggesting that, when observing actions and inferring intentions of others, interoceptive signals processed by the IC are indispensable reference information (Jabbi et al., 2008). Although infants are still in the prespeech stage in the first year, they have developed extensive emotion recognition based on internal arousal. Studies have shown that infants aged 7–12 months can distinguish basic emotional categories such as happiness, sadness, anger, fear, and disgust through classic facial expressions and actions (Ruba et al., 2017; Safar and Moulson, 2017). Through interoception, children can understand the emotional actions of other people. When children are less than 2 years of age, they can match emotional actions of others with related events (Ruba et al., 2020). For example, Ruba et al. (2019) used three emotions with the same valence and arousal, namely anger, disgust, and fear, and tested 14- or 18-month-olds to observe specific events and emotional expressions of performers. The results showed that infants could match negative emotions with specific events. However, understanding these different negative emotions may have different developmental trajectories.

The functional connection between the IC and other brain regions, such as the fronto-temporal network, enables children to consciously process interoceptive signals and integrate their feelings and cognition into the understanding the actions of others (Adolfi et al., 2017), so as to provide an internal reference to infer action intentions or goals of others. Interoception of children develops rapidly from early implicit perception to later sub-components such as interoceptive accuracy, awareness, sensitivity, etc. (Palmer and Tsakiris, 2018). According to the suggestion of Garfinkel, interoception components have different neural developmental trajectories (Garfinkel et al., 2015b). Among them, interoceptive awareness is closely related to metacognitive function, which is a high-level “metacognitive” knowledge of interoception and is affected by the neural development of the anterior cingulate cortex, prefrontal cortex, and other brain regions (Garfinkel et al., 2015b). Thus, it matures later and seems to develop during childhood and throughout adolescence (Klabunde et al., 2019; Bonaz et al., 2021).

### The Application of Action Understanding and Interoception Model

The current model describes the typical development of action understanding and interoception in children. However, individual differences of children could affect the developmental relationship between action understanding and interoception. For example, temperament discrepancy of infants shows in reactivity to stimuli and self-regulation (Stifter and Jain, 1996). The behavioral response of early infants to stimuli is mainly biologically driven, which is closely related to interoception. So the individual difference in interoception affects the early action development. Porges et al. (1994) measured the relationship between the behavioral reactivity and autonomic state in 9-month-old infants. They found that high cardiac vagal tone was associated with greater behavioral reactivity. In terms of regulation, a longitudinal study of Stifter and Jain (1996) suggested that 5-month-old infants with high vagal tone showed more regulatory behavior at 18 months of age.

Based on the model of action understanding and interoception of children, infants establish a behavioral response association through interoceptive information and feedback in early stage. Infants with more active autonomic nervous system may produce more and possibly conflicting interoceptive cues simultaneously (Porges et al., 1994; Stifter and Jain, 1996; Huffman et al., 1998). When more activated infants understand actions, they need to pair multiple interoceptive stimulus and response signals, so as to slow down learning or confuse action understanding. This may explain that infants with interoception individual differences perform different in behavioral regulation and interaction with caregivers. For example, according to the report of a mother, a 9-month-old infant with high cardiac vagal tone has more difficult in temperament (Porges et al., 1994). Five-month-old more activated infants showed more regulatory behavior at 18 months (Stifter and Jain, 1996). The regulatory behavior affects the interaction between infants and others. For example, 5-month-old highly activated infants have shown the employment of regulatory strategies when interacting with others at 14 months of age (Fox, 1989).

Children with different temperament types have differences in autonomic nervous system stimulation and information feedback, which affect action understanding development of an infant. In addition, congenital aphantasia with abnormal sensory imagery may also affect action understanding of children. Sensory imagery refers to a perceptual representation present in mind, but the stimulus is not actually being perceived (Kosslyn, 2005). Sensory imagery depends on perceptual representation and activates the corresponding cerebral cortex, so as to produce vivid image and experience. For example, action imagery activates the human motor cortex (Porro et al., 1996; Dechent et al., 2004). Without stimulation, aphantasia cannot produce corresponding imagination and representation in mind (Zeman et al., 2015). In approximately 1–3-year-olds, body action representation plays an important role in infant action simulation. After observing the actions of others, children need to mirror actions in mind. However, individuals with multimodal congenital aphantasia cannot imagine sensations that are generated through interoception, such as representations of emotional states or experiences which depend on somatosensory and insular sensations (Wicken et al., 2021). Therefore, the defect of action imagery may delay the children with congenital aphantasia to simulate and understand the actions of others.

The nerve defect of aphantasia involves not only the corresponding sensory region, but also other brain regions. For example, Hassabis et al. (2007) found that sensory imagery involves a wide network, including ventromedial prefrontal cortex, hippocampus, posterior parietal cortex, etc. When children with congenital aphantasia observing action have problem in action representation, the compensatory nervous system may aid to generate good action models. First, in visual imagination of action observation, Keogh and Pearson (2018) found that congenital aphantasia has a defect in low-level visual imagery, mainly in visual details rather than spatial relations. This provides the possibility for children to observe and simulate the spatial information of action. Second, there is no unique mental imagery cortical network (Mellet et al., 1998). Mental imagery has a high degree of interaction with other cognitive functions, such as situational memory and executive function. With the growth of memory and experience of children, retrieving memory information through situational cues can trigger related emotional and physiological experiences (Moulton and Kosslyn, 2009). Top-down processing could further improve perception dependence through enhancing the information exchange between different brain regions (Moulton and Kosslyn, 2009; Dawes et al., 2020). Therefore, children with congenital aphantasia may delay their action understanding development due to the imagination defect, but cognitive functions improvement plays a compensating role.

The current research takes temperament and congenital aphantasia as an example to explore the practical model application of action understanding and interoception of children. The model predicts the impact of temperamental individual differences on the simple association of action understanding and interoception in infants. When observing and simulating action, action imagination defect may cause developmental delay. According to the model of action understanding and interoception of children, on the one hand, through interoception development characteristics, such as interoceptive accuracy or interoceptive sensitivity, we can predict action development of typical children. On the other hand, physiological mechanism deficiency causes the abnormal development of action understanding and interoception. For example, if the nerves controlling muscles and tendons lack PIEZO2 protein, individuals will lose proprioception and perform uncoordinated actions (Dance, 2020). Therefore, when children are found to have abnormal development of interoception, timely intervention and guidance should be taken.

## Conclusion

This review systematically explored how interoception promotes the development of action understanding of children. At different stages, there are substantial differences in the role that interoception plays in promoting action understanding of children, the neural mechanisms of which provides a physiological basis for development. However, the current model of action understanding and interoception of children needs more support evidence from empirical studies. For example, future research can focus on the internal neural mechanism of observing and simulating actions in children with congenital aphantasia.

## Author Contributions

HZ wrote the article. QG provided suggestions and guidance for the article. WC conceived the structure of the article. QW revised the article. All authors contributed to the article and approved the submitted version.

## Conflict of Interest

The authors declare that the research was conducted in the absence of any commercial or financial relationships that could be construed as a potential conflict of interest.

## Publisher’s Note

All claims expressed in this article are solely those of the authors and do not necessarily represent those of their affiliated organizations, or those of the publisher, the editors and the reviewers. Any product that may be evaluated in this article, or claim that may be made by its manufacturer, is not guaranteed or endorsed by the publisher.
